# Cultured Meat on the Social Network Twitter: Clean, Future and Sustainable Meats

**DOI:** 10.3390/foods11172695

**Published:** 2022-09-03

**Authors:** Lucie Pilařová, Lucie Kvasničková Stanislavská, Ladislav Pilař, Tereza Balcarová, Jana Pitrová

**Affiliations:** Department of Management, Faculty of Economics and Management, Czech University of Life Sciences Prague, 165 21 Prague, Czech Republic

**Keywords:** cultured meat, cultivated meat, clean meat, future meat, sustainable meat, alternative protein, Twitter, social media analysis

## Abstract

The rapid development of technologies for cultured meat production has led to new challenges for producers regarding appropriate communication with future customers in order to deliver products to a viable market. Communication analysis of social media enables the identification of the key characteristics of the monitored topic, as well as the main areas of communication by individual users based on active digital footprints. This study aimed to identify the key characteristics of cultured meat based on communication analysis of the social network Twitter. Communication analysis was performed based on 36,356 Tweets posted by 4128 individual users. This analysis identified the following main communicated characteristics: clean meat, future meat, and sustainable meat. Latent Dittrich allocation identified five communication topics: (1) clean and sustainable products, (2) comparisons with plant-based protein and the impact on agribusiness, (3) positive environmental aspects, (4) cultured meat as an alternative protein, and (5) the regulation of cultured meat.

## 1. Introduction

Food is an important part of everyday life and, for most people, is no longer just about survival. Instead, food choice is a social-cultural decision that is influenced by both long-term and short-term factors [1]. Food choices affect physical and mental health, as well as our self-perception and how we relate to our surroundings in terms of our nutritional trends, our relationship with the environment, and animal welfare [2,3]. One significant and steadily growing consumer trend is the market for vegan products, which includes food, cosmetics, clothing, and entertainment [4,5]. This trend includes the responses of companies supplying vegan products and services to satisfy the market, where customers are increasingly interested in the effects of products on their health and the impact of consumption on society and the environment [6]. Companies produce vegan products not only to boost sales, but also to strengthen their market position by combining these products with their corporate social responsibility (CSR) activities; moreover, they communicate the impact of these products on environmental, ethical, and social responsibilities to interested groups [7].

In parallel, there is an important trend in agricultural production, including meat production, to create more environmentally friendly products [8]. Agriculture is responding to a growing interest in the environment using comprehensive approaches, such as organic agriculture [9]; individual products in the field of meat substitutes, such as “meat” based on hydrolyzed vegetable protein [10]; and cultured (cultivated) meat technology, which uses innovative technology to provide high-quality protein that is healthy, bacteriologically safe, and friendly to animals, with a relatively small ecological footprint [11,12,13,14].

Cultured meat aims to elicit beneficial effects for animal welfare and the environment by reducing the need for water, land, and energy [15,16]. Specifically, its production requires 7–45% less energy use, 99% less land use, and 82–96% less water use, and generates 78–96% fewer greenhouse gas emissions (depending on the product) [15,17].

To ensure the success of cultured meat, it is necessary to focus not only on the production technology but also on the marketing of the products, and the matter of establishing an appropriate marketing strategy to communicate with customers [18]. A key factor in this process is the identification of social media communications that influence future consumers’ perceptions of the products [19]. Companies focused on the production of cultured meat realize the importance of marketing, and have started developing marketing strategies to reach potential customers [20,21]; accordingly, potential customers are reacting to these messages, and this communication can be analyzed.

This research aimed to identify the key characteristics of cultured meat through communication analysis of the social network Twitter.

## 2. Theoretical Background

Cultured meat, also described as cell-based, cultured or cell-cultured, lab-grown, in vitro, artificial, synthetic, clean, or slaughter-free meat [16,22,23,24], shows significant promise as a future source of animal protein [25,26]. Cultured meat does not require the use of slaughtered animal carcasses to produce meat products, opening up the possibility of food proteins becoming less reliant on traditional animal agriculture, which has detrimental impacts on the climate and on livestock welfare. Traditional animal agriculture also generates products with an increased likelihood of containing foodborne pathogens, and has lower overall efficiency [16,27]. Cultured meat could also serve as a meat analog that requires no input from living animals. Future innovation could reduce the need for animal involvement entirely, thus making cultured meat a viable consumption option for people who identify as vegetarian, vegan [28,29], and flexitarian [30]. Previous studies have identified continual growth in the plant-based meat market across the world [31,32,33,34], which may hamper cultured meat from reaching the meat alternatives market [35]. Another issue is the perception of cultured meat by vegans; surveys have shown that vegans are more likely not to pay for cultured meat [36,37].

The consumption of meat and animal products has long been a cornerstone of the human diet. A previous study [38] reported that meat production has tripled over the past 50 years. However, findings from another study [16] indicate that, although meat consumption is projected to continue increasing, the price points for meat are also expected to increase because of inefficiencies in global livestock production systems, including limited opportunities for innovation, decreasing availability, and the consequential increasing costs of resources including land, energy, and water. With meat consumption already on the rise, one study [39] noted that, considering the world population is projected to expand to 9.7 billion people by 2050, food production will have to increase by at least 70% (and probably more) to keep up with demand and enhance global food security. Therefore, there is a growing need to identify new sources of meat production, because traditionally produced livestock has the potential to become prohibitively expensive in the future. Furthermore, analysis of the livestock industry through the lens of economic cost ignores another tangible cost: the impact on the environment and climate. With regard to greenhouse gas emissions [27], one previous study [40] noted that the size of the global middle class is rapidly expanding, leading more consumers in developing countries to have an interest in and the means to purchase animal products, including meat; consequently, consumer demand and global food security needs are exerting combined pressures on the livestock industry to produce greater amounts of animal products that are “environmentally sound, socially responsible, and economically viable”. 

Recent innovations and trends in the alternative protein industry have led to the rise of plant-based imitation meat products [16]. However, even with the increasing acceptance and popularity of plant-based meat, traditional meat products remain a cornerstone of many people’s diets. This enduring desire to eat meat and other animal-based products is a major factor that drives innovation in cellular agriculture research into cultured meat. Other factors include the finding that the consumption of traditional meat has adverse effects on human health. One study [28] reported that a growing body of evidence links the consumption of meat, particularly red meat and processed meat, to a variety of chronic illnesses, as well as an increased risk of early mortality. This study [28] noted the potential for the nutritional value of cultured meat to exceed that of traditional meat, considering that some studies [41] argue that the levels and types of fat (i.e., more similar to the “good” fats found in fish and nuts, as opposed to “bad” saturated fats) in a product can be adjusted. Another study [42] noted that the conditions under which cultured meat is produced can be significantly safer than those associated with traditional meat production due to decreased exposure to pathogens; the resulting decreased need for antibiotics will reduce the probability of animal diseases spreading to humans and also reduce the rise of antibiotic resistance in the human population. 

Although many findings imply that cultured meat will have a prosperous future, all new technologies pose a certain level of risk, and the possibility of adverse impacts resulting from a shift away from traditional livestock production should also be considered. Rapid cell growth and division during the propagation of cell lines could result in a higher risk of mutations and the growth of potentially cancerous cells [25,43]. While cancerous cells can develop in cultured meat, they would be dead and no longer replicating by the time consumers digest them, meaning long-term harm to consumers is unlikely, although further research is needed [43]. The traditional meat industry creates and exacerbates health risks for its workers, mostly in terms of respiratory illness and infection [28,44]. Furthermore, communities located near meat-packing plants are more likely to develop respiratory illnesses, become infected with animal pathogens, including those for which existing antibiotic interventions are obsolete, and report higher levels of stress and adverse effects on mental health [28,45].

Another angle from which to analyze the benefits of a shift away from traditional meat production and towards a cultured meat system is not through the lens of human health and welfare, but in terms of the health and welfare of domestic and wild animals [28,29]. Such a shift will have major ramifications for the recognition of animals, both domestic and wild, as subjects rather than objects [16]. The traditional livestock industry has historically had an adverse impact on a range of different ecological factors, including the environment, animal welfare, and global food systems [16,25,28,46]. Livestock production is massively detrimental to efforts to prevent global warming. One study [46] found that reducing meat consumption is essential if we are to remain below the projected global temperature increase of 1.5 °C. Another study [28] found that the largest percentage of greenhouse gas emissions comes from some of the most popular types of meat produced for consumption, namely, ruminant animals (cows and goats) and crustaceans, particularly shrimp and lobster. Meanwhile, cultured meat production leads to a lower immediate level of greenhouse gas emissions than conventional meat production. If a food system based on cultured meat is extrapolated over centuries, cultured meat will likely cause the planet to accumulate more CO_2_ than would traditional livestock [47]. In comparison with traditional meat, cultured meat requires fewer ecological resources to produce and has less of an impact on the environment [48]. Another study [16] found that cultured meat requires 99% less land and 98% less water than beef production, and 66% less land and 92% less water than chicken, turkey, goose, and duck production. Additionally, the scientific production of meat has ramifications outside the food industry. Humanity is reliant on animal products for a variety of uses besides consumption for food and nutrition, including biomedical therapies and technologies that are reliant on the cultivation of tissue for experimentation, research, and production [28].

Based on previous studies and past waves of technological innovation, even in this area, processes such as material production will be concentrated in developing countries, have lower economic value, and use less skilled working populations [16]. The accumulation of capital and power is possible not only for developed countries but also for existing transnational food systems, which already have a high level of control over the global food system [25]. On the micro level, a shift to cultured meat could harm local meat producers and farmers, and heighten rural–urban divides [49]. One study [50] projected that plant-based protein and cultured meat will be five times cheaper than traditional animal products by 2030, resulting in a massive market shift that could result in the loss of half of the current beef and dairy production jobs, and a 40–80% reduction in the value of farmland in the United States alone.

### 2.1. Attitudes toward Cultured Meat

Current attitudes toward cultured meat vary. One study [29] found that, while most people are willing to consume cultured meat, there is greater interest among the younger generation, highly educated people [18,34], and people from developed countries [51]. Linguistic choices surrounding cultured meat are a significant predictor of people’s willingness to potentially engage with the product. Another study [40] found that over half of the people surveyed believed that the word “meat” should not be used to describe cultured meat, and that the descriptor used to modify the word “meat” could have powerful effects. Similarly, a study [16] that analyzed the various terms used to describe cultured meat reported that “process-centered terms” such as “cell-based”, “cultured”, and “cultivated” were considered to be the most transparent and “free of moral judgment”, while terms such as “lab-grown”, “in vitro”, and “synthetic” were negatively associated with artificiality in consumers’ minds. Other research [40] reinforced these findings, noting that, when consumers perceive a product as more “high tech”, they are more suspicious of it and less inclined to purchase or consume it.

Attitudes in society vary, with some consumers believing that cultured meat is unethical because it is “unnatural” and claiming that producing it is akin to “playing God” [52]. Attitudes toward the “unnatural” and “unethical” aspects of cultured meat production often translate into distrust of cultured meat producers and future government regulation, with some consumers reporting fears that they may consume cultured meat in the future without their knowledge or consent [52]. These concerns are particularly prevalent in online and social media discussions of cultured meat [52]. 

Other common concerns about cultured meat center on the perceived cultural changes in a society that is less strongly based on traditional livestock production, which may adversely impact traditional farmers [49] and influence certain meat-centered rituals, such as barbecues, while Thanksgiving turkeys would cease to exist in their current form [52,53]. However, consumers also recognize the potential for cultured meat to achieve other significant changes in terms of alleviating human and animal suffering [54,55], to provide protein to low-income communities, and to vastly improve animal welfare conditions worldwide. According to one previous study, animal welfare was the principal reason given for supporting cell-based meat [29]. The most significant factors driving perceptions of cultured meat are price [42,52], taste, and texture [39].

### 2.2. The Future of Cultured Meat

Owing to high-profile investments in start-up companies focused on producing cultured meat, optimism is high about the future of this industry. However, one study [25] has cautioned against overexcitement. As with any emerging technology, a great deal of media and investor attention focuses on the industry in its infancy. However, as research and development continue, and producers run into problems regarding scale, efficiency, and distribution, the initial excitement and funding offers wane, and the pace of innovation slows significantly. The advent of the COVID-19 pandemic may have accelerated interest in the industry, considering the ways in which cultured meat will lower the risk of pathogen transmission from domestic animals to humans, possibly preventing future pandemics, as well as the potential of cultured meat to bolster the supply chain and empower local economies to produce their own meat products instead of being reliant on a global meat production structure that can be easily disrupted [40,56]. One study [16] speculated that cultured meat could be imminently available, while another [57] projected that the widespread introduction of cultured meat will not occur for another 5–10 years, with plant-based meat retaining the majority of the protein alternatives market until at least 2030. The possibility of producing other cultured animal products, such as milk and eggs, as well as further development of tissue engineering, is paving the way for greater acceptance of cultured meat [25,58].

Another potential barrier to the widespread introduction of cultured meat is the extremely technical nature of its production, which requires employees who are highly educated and specialized in terms of their training and expertise. One study [25] noted that, because of the uncertainty surrounding exactly how and where cultured meat will be produced, there is also ambiguity around future government support for the industry through grants, subsidies, and training programs. Other key questions will have to be answered before cultured meat becomes as commonplace as traditional meat [25]. Will cultured meat production be the purview and industry of bioscientists, traditional farmers, or large-scale agribusinesses? Which sector is already empowered to adopt the technology, and which sector will this technology primarily benefit (i.e., who will reap the majority of the profits) [59]? Will cultured meat be produced primarily in the global north or south? What will the long-term political, social, environmental, and ethical consequences of large-scale cultured meat production be, and what impact will these consequences have on people of various socioeconomic backgrounds and nationalities? What are the possible ways to reach the global market? How should information about cultured meat be communicated, and how can its correct positioning be identified? While the potential benefits of cultured meat are powerful motivators, the industry has a complicated and uncertain future.

This article contributes to the identification of relevant communications on social networks, and provides an important input analysis for the correct positioning of cultured meat.

## 3. Materials and Methods

Latent Dittrich allocation (LDA) [60] and the Framework for Social Media Analysis Based on Hashtag Research (SMAHR) [61] were used to analyze communications about cultured meat on the social network Twitter. 

LDA is the most representative topic model based on whole text in Tweets. It is a three-level hierarchical Bayesian model [60], an unsupervised machine learning technique used to generate a representation of a document (in this case, Tweets) by topic extraction. Based on extraction, each topic consists of a set of words, which represent the meaning of the topic. For this analysis, the Python Gensim module was used [62]. LDA topic exploration has previously been used in studies of occupational differences in reactions to the COVID-19 pandemic [63], health informatics [64], the United States presidential election [65], and online food delivery [66].

SMAHR is a framework based on hashtag research that focuses on a specific part of Tweets: namely, hashtags. A hashtag is a communication element that begins with the symbol “#”. Hashtags have two key functions on social media platforms. The first is to filter posts, with social media site algorithms providing an archive of messages connected to a hashtag [67]. The second is to highlight values, experiences, attitudes, and opinions in the message [68,69,70,71]. In the case of cultured meat, the hashtag “#cleanmeat” can be used to highlight clean meat characteristics. SMAHR has previously been used in studies of organic foods [72], farmers’ markets [73], sustainability [19], CSR [74], and gamification [75]. 

Based on the combination of LDA and SMAHR, the data analysis process consisted of the following four steps:

(1) Data acquisition: the Twitter API [76] was used to extract messages (Tweets) from the Twitter database. Data were collected between 1 January 2005 (Twitter API limitation) and 30 June 2022. Tweets were captured by the Python Script [77] based on the following condition: all Tweets including [“#culturedmeat” OR “#cultivatedmeat” OR “#cellbasedmeat” OR “cultured meat” OR “cultivated meat” OR “cell-based meat” OR “cellbased meat”]. During that period, 36,356 Tweets were captured from 4128 unique users. This exported dataset contained all Tweets sent to the Twitter social network that included the selected topics [“#culturedmeat” OR “#cultivatedmeat” OR “#cellbasedmeat” OR “cultured meat” OR “cultivated meat” OR “cell-based meat” OR “cellbased meat”] during the monitored period.
(2) Content transformation:
  (a) SMAHR section. Because this framework was designed exclusively for hashtags, any terms that were not preceded by the hashtag symbol (“#”) were removed. Consequently, the dataset included only hashtags. Thereafter, all uppercase characters were converted to lowercase letters in order to avoid duplication (e.g., the computer may interpret #meat, #Meat, and #MEAT as three distinct hashtags). The final modification was to separate strings of connected hashtags, such as “#meat#innovation”, which was converted to “#meat; #innovation”. The dataset was then loaded into Gephi 0.9.3, and a hashtag network based on hashtag interdependence was created. Gephi is an open-source graph and network visualization and exploration software [78].
  (b) LDA section. The dataset was converted to a CSV (comma-separated value) format.
(3) Data mining: for communication analysis, the following data-mining methods were used:

### 3.1. SMAHR Section

(a) **Frequency:** a frequency is a number that describes the frequency of hashtags in a network.
(b) **Eigenvector centrality:** an extension of degree centrality that measures the impact of hashtags in a network. Eigenvector centrality is determined based on the assumption that connections to hashtags with high-degree centrality values have a greater effect than connections to hashtags with similar or lower-degree centrality values [79]. A high eigenvector centrality value indicates that a hashtag is linked to a large number of hashtags with high-degree centrality values. Eigenvector centrality was determined using Equation (1):
  (1) $$x_{v}=\frac{1}{\lambda }\underset{t\in M\left(v\right)}{\sum }x_{t}=\frac{1}{\lambda }\underset{t\in G}{\sum }a_{v,t}x_{t}$$
  where *M*(*v*) denotes a set of adjacent nodes, and λ is the largest eigenvalue. Eigenvector *x* can be expressed by Equation (2):
  (2) Ax=λx
(c) **Communication peak analysis:** this analysis aimed to identify peaks based on the frequency with which Tweets were sent. Peaks were identifying based on the following Equation (3):
  (3) $$\tau =\frac{n_{t}}{Øn_{3}}$$
  where:
  (4) $$Øn_{3}=\frac{n_{t-3}+n_{t-2}+n_{t-1}}{3}$$
  where n_t_—the number of messages in the evaluated month. Ø_n3_—the average number of messages for the previous 3 months. τ—the threshold value for determining the peak (peak = τ > 5).

### 3.2. LDA Section

(a) **Topic modeling:** LDA is a three-level hierarchical Bayesian model [60], an unsupervised machine learning technique used to generate a representation of a document (Tweets, in this case) by topic extraction. Based on extraction, each topic consists of a set of words, which represent the topic’s meaning. For this analysis, the Python Gensim module was used [62].

(4) Knowledge representation: knowledge representation is the process of applying visualization tools to represent the results of data mining. To represent knowledge, a synthesis of key attributes and outputs from the data assessment process is used.

## 4. Results and Discussion

**Figure 1:**
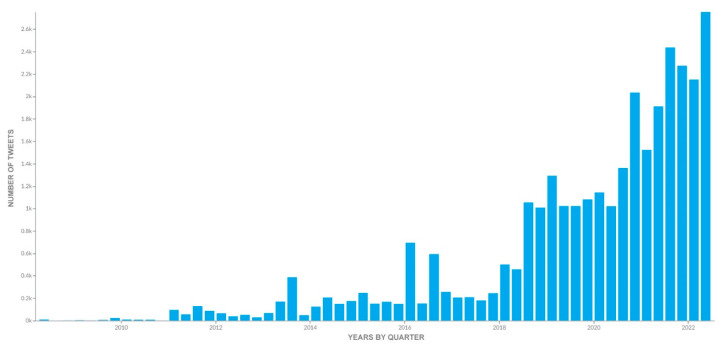
Number of tweets mentioning cultured meat from 1 January 2005 to 30 June 2022. The figure shows the continuous increase in cultured-meat-based communications on the social network Twitter.

Data were collected between 1 January 2005 and 30 June 2022. During that period, 36,356 Tweets were captured from 4128 unique users. There was an annual increase in communications related to cultured meat (Figure 1).

The first Tweet mentioning cultured meat was written on 13 April 2008: “What if you can have your meat, be ethical, and environmental, too? Manufactured Meat, baby! (cell cultured meat, in point of fact.)” [80]. This Tweet has not received any likes, no users have shared it, and no one has commented on it.

**Figure 2:**
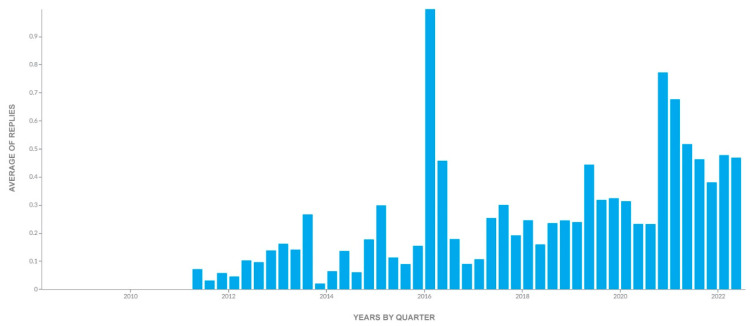
Average numbers of replies to Tweets mentioning cultured meat from 1 January 2005 to 30 June 2022. The figure shows the reply peaks in cultured-meat-based communications on the social network Twitter.

Social media users started replying to this topic as early as 2011. The average number of replies was highest in 2016 (Figure 2). This was due to the Tweet “If cultured meat is molecularly identical to beef, pork, etc., and tastes the same, will you switch to eating it?” [81]. A total of 406 users commented on this Tweet, and, in the voting function, 14,614 users voted, with 83.4% voting “Yes” and 16.6% voting “No”. 

The Tweet from 2018 “It may not happen at large scale, but a micro-brewery type of system for Cultured Meat is plausible” received the most favorites (4775) [82]. Thus, this topic resonates among users of the social network Twitter.

Table 1 shows the frequencies of individual hashtags in Tweets.

The hashtags ranked first (#culturedmeat), second (#cultivatedmeat), and seventh (#cellbasedmeat) included the most commonly used synonyms of this product. The most frequently used terms for this product are “cultured meat”, “cultivated meat”, and “cell-based meat” [16,83]. Other frequently used synonyms were found in the hashtags ranked fourth (#cellag) and eleventh (#cellularagriculture), which reference cell-based meat. A study [84] reported that the term “lab grown meat” (#labgrownmeat) is mostly applied for marketing purposes, because the future commercial application of cellular agriculture will not happen in a laboratory. A current challenge [85] concerns the negative link between the term “lab grown meat” and a positive perception of cultured meat. This is a negative perception of all aspects of cultured meat, except for animal friendliness. An identified area of great significance is associated with the hashtag “#plantbased”, which refers to plant-based meat, i.e., plant-based meat alternatives that are made from plant extracts and/or plant components that imitate and replace meat [85]. This area was communicated mainly in the field of partnership meat alternatives such as fermented meat substitutes, plant-based meat, and cultured meat. For example, “From major investments in the plant-based supply chain to a partnership between precision fermentation and cultured meat start-ups”, “2021 was a breakthrough year for #plantbased foods, #cultivatedmeat and #fermentation”, and “We’re launching a Good Food Institute Europe newsletter early next year! The newsletter will be your guide to the major milestones in the continent’s #plantbased, #cultivatedmeat and #fermentation sectors”. An important factor is the nonpolarization of individual areas. Plant-based meat is defined as a better choice in the field of meat alternatives, and this can be used in the field of technological synergy and in the marketing communications and market positioning of meat alternatives as substitutes.

Another important hashtag in the top 20 most frequently used was #vegan, which refers to a vegan lifestyle. This included communication about the possible inclusion of cultured meat in vegan diets. Example Tweets included “Would you eat lab-grown meat? It’ll take some time to see if the benefits are positive in terms of health and the environment, but these innovations are a step in the right direction! #cultivatedmeat #climatechange #vegan #alternativeprotein” and “One of the ugly truths of conventional meat production. Switching to #cultivatedmeat means no more lives will be taken to feed humans. We can end the cruelty! #vegan”. This raises an important question for further research. What type of vegan (people who choose to be vegan for health, ethical, environmental, or religious reasons) is a potential consumer of cultured meat? 

### 4.1. Eigenvector Centrality

When analyzing eigenvector centrality, three main hashtags were identified: namely, “#culturedmeat”, “#cultivatedmeat”, and “#cleanmeat”. (Table 2) Based on eigenvector centrality values, clean meat was the main communicated value. Compared with frequency analysis, different hashtags were identified in the top 20, including “#innovation” and “#science”. The hashtag “#innovation” refers to sustainable innovation. An example Tweet was “In the meantime, a throwback to a past edition of our visual blog, focusing on #CleanMeat and #sustainable #innovation in the #foodindustry”. The hashtag “#science” refers to the scientific research background of this product.

### 4.2. Communication Peak Analysis

Based on the τ > 5 threshold, six peaks were identified.

(1) **February 2011**—a peak that is tied to a comment made by the biologist Vladimir Mironov, who came forward with the statement that cultured met is an “inevitable and inescapable” technology in the light of increasing population growth [86].
(2) **June 2011**—this peak was started by a report from the University of Oxford titled “Lab-grown meat would ‘cut emissions and save energy’, kde vyšla studie, která tvrdila, že “Cultured meat’, or meat developed using tissue engineering techniques, will produce 96% fewer greenhouse gas emissions than conventionally produced meat, according to a new study” [87].
(3) **August 2013**—this peak was started by a taste test and cooking demonstration in London, United Kingdom, in August 2013. Dr. Mark Post from the Netherlands created the first hamburger made from cow stem cells for USD 325,000.
(4) **February 2016**—this peak was started by Upside Foods, when it achieved two main goals. (a) Upside Foods produced cultured poultry for USD 20,000 per kg. (b) Produced cultivated beef (in form of meatball) for USD 40,000 per kg.
(5) **July 2016**—following their previous success, Upside Foods decreased their per-kilogram production costs to USD 5280.
(6) **December 2020**—The Singapore Food Agency (SFA), the city-principal-state’s agency for food-related concerns, authorized the sale of a cultured meat product. It was the world’s first approval of cultivated meat sales.

### 4.3. Regional Differences

Cultured meat research is spread across the globe, with a total of 107 major players worldwide. Most companies are in North America (32) and Europe (29) [88]. Based on this, the comparison of two regions focused on (1) the United States of America and (2) the European Union (including the United Kingdom, which left the EU on 31 January 2020).

Of the total number of 36,356 Tweets, 63% (22,904 Tweets) contained geolocation information, from which it was possible to determine that 4396 Tweets were sent from the USA and 2034 Tweets from the EU.

From the outset, it is necessary to point out that this is an analysis that is based on a small sample size. 

Based on the results, it can be determined that, in the USA, there is a greater tendency to communicate the importance of cultured meat for the entire industry and its production possibilities for the market. On the other hand, the EU communicates a forecast related to the possibility of replacing traditional meat with cultured meat. However, in general, similar main values of these communications can be identified (for more details, see Table 3).

### 4.4. Community Analysis

**Figure 3:**
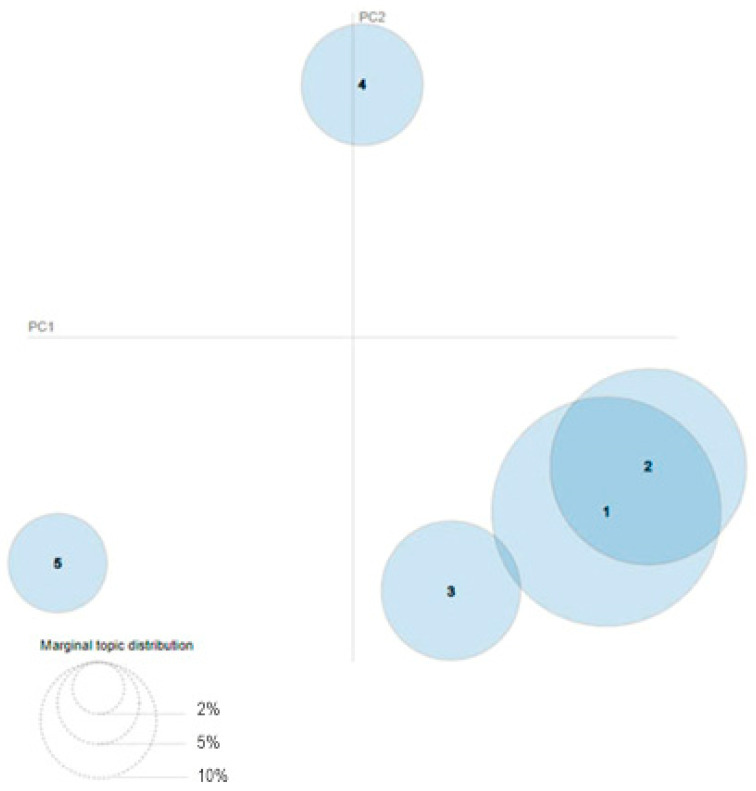
Intertopic distance map. The figure shows the relative position and size of five main topics in the area of cultured meat on the social network Twitter.

Based on LDA exploration, five communities were identified (Table 4). The relative position and size of each topic are displayed in Figure 3.

The largest community was called “Clean and sustainable product” according to the key terms. This community focused on communicating the main values of cultured meat, which include sustainable and clean meat production and the growth of a food industry without animal cruelty. It accounted for 39% of communications. 

The second-largest community was called “Comparison with plant-based protein and impact on agribusiness”. It focused on two basic areas: namely, the future of the food market, with a focus on the impact of cultured meat on the global agriculture business, and the replacement or supplementation of plant-based protein. This discussion is very important because cultured meat is practically independent of crop agricultural production, whereas plant-based meat is directly linked to plant agricultural production. These challenges were discussed in this community. It accounted for 28.4% of communications. An example was the Tweet: “This wannabe hot-take unwittingly made a case for #plantbasedmeat vs “cultured” meat, as plant-based relies on farmers in the supply chain. Whereas cultured could, more or less, eliminate farmers”. 

The third-largest community focused on the positive environmental aspects of cultured meat. Its lower energy consumption and effect on greenhouse gas emissions, which are confirmed by research, were the most communicated aspects [15,17]. It accounted for 14.4% of communications.

The fourth-largest community was called “Cultured meat as alternative protein”. It focused on communicating that cultured meat is a source of better and alternative protein. Veganism was also widely discussed. This area is very important because food choice preferences are reflected through market trends, and a major, steadily growing trend in consumption is the vegan market, which includes not only food but also cosmetics, clothing, and entertainment [4]. Being vegan is a growing philosophy, based on a way of life that seeks to eliminate all forms of animal exploitation and cruelty for food, clothing, or any other purpose [89]. This influences the market because companies are trying to meet the needs of this emerging market by providing vegan products and services, with customers becoming increasingly interested in the effects of products on their health and the impact of consumption on society as a whole [6]. Companies that produce vegan products not only seek to increase sales but also to strengthen their market position by linking these products to their CSR activities, and they communicate the environmental impact of these products for ethical and social responsibility [7]. This raises a question for further research: is it possible to include cultured meat in the portfolio of meat producers as a CSR product, to create a positive relationship with the public?

Current research defines four main motives for veganism [90]. First, an ethical motive, i.e., people who choose a vegan lifestyle because they are against animal cruelty. An ethical vegan does not want their life to involve animal exploitation. Second, an ecological motive, i.e., people who become vegans to live a “greener” lifestyle in an effort to improve the ecology of the planet. Third, a health motive, i.e., people who become vegans for health reasons, because they believe that a vegan diet is healthier than a traditional diet; for example, the consumption of red meat increases the risks of colon cancer and heart disease. Fourth, a faith-based motive, i.e., people who choose a vegan diet based on their spiritual beliefs. An example is Jainism, which is characterized by the consumption of a strict vegan diet. This raises a question for further research: what types of vegans are potential consumers of cultured meat?

The current food market is influenced by food choice trends. Research conducted in the UK confirmed that even flexitarians and omnivores purchase vegan products [91]. Thus, there is a trend whereby many people consider vegan food to be part of a healthy diet but do not want to be vegetarians. This tendency was also identified in a social network study, which reported that veganism is associated with the hashtag “#healthyfood” [5]. Not only are researchers responding to this trend by analyzing the determinants that influence the purchasing of vegan food in different countries [92,93], but food producers are also adapting to these new trends: for example, by producing plant-based meat. Flexitarians are the most important market for these products [30]. This raises a question for further research: is cultured meat a suitable product for flexitarians? 

The fifth-largest community was called “Regulation of cultured meat”, which focused on regulation of cultured meat technology by the US Department of Agriculture (USDA) and the United States Food and Drug Administration (FDA), which announced joint regulation of cell-cultured meat. An example of this type of communication was the Tweet: “USDA and FDA announce joint regulation of cell-cultured meat”. This area accounted for 7.2% of communications.

These results are in agreement with a study that focused on news media [94]. This analysis identified six basic areas: (1) benefits, which are possibly represented in our study by the topics “Clean and sustainable product” and “Positive environmental aspects”; (2) history, which, in our research, did not create its own community, but is represented by the peaks that identify the main milestones in the field of cultured meat; (3) current livestock production problems, which can be found in our research in the topics “Comparison with plant-based protein and impact on agribusiness”; (4) process areas were identified in the news media study, with a focus on production technologies; this is an area that can be found in the topic “Comparison with plant-based protein and impact on agribusiness”. The last area identified by news media analysis was (5) skepticism, which was not identified in this study as a main area of communication on Twitter.

This study thus confirmed four out of five areas identified based on an analysis of news media. On the other hand, our analysis identified the area “Regulation of cultured meat”, which was not found in the news media analysis. These results support the necessity of triangulation of individual methods to obtain a holistic view of individual areas, and the importance of using various methodological tools in the field of communication analysis.

## 5. Limitations of the Research

The first limitation of this research is that only one social network, Twitter, was used. This is due to a restriction on downloading data via the Instagram API [95] introduced by Meta in 2018, after the Cambridge Analytica data scandal.

The second limitation of this study is the “Regional differences” section, which was created based on only 6430 Tweets. This is due to a combination of the declining number of users sharing geolocation information, and the fact that cultured meat remains a relatively new topic.

Social media analysis provides an opportunity for the construction of timely socio-economic indicators. Despite the numerous benefits of researching social media for this purpose, there are certain statistical and quality concerns [96]. Similarly to traditional media, such as newspapers, television, and radio, there may be a certain political or business interest in this area that will try to influence public opinion. The triangulation of different methods should prevent this phenomenon. This research, which focused on the analysis of social media, is thus an important part of other studies that deal with the analysis of other communication channels.

## 6. Future Research

In the Results and Discussion section, three questions were identified for further research:

(1) Is it possible to include cultured meat in the portfolio of meat producers as a CSR product to create a positive relationship with the public?
(2) What types of vegans are potential consumers of cultured meat?
(3) Is cultured meat a suitable product for flexitarians?

Furthermore, due to the relative novelty of the topic, it would be appropriate to make a regional comparison between the regions of Africa and the Middle East, Asia Pacific, Europe, Latin America, and North America, once there are more Tweets on the topic. Given the current trend, it would be possible to reach 200,000 tweets over the next 3–5 years.

## 7. Conclusions

Communication analysis of Twitter identified the main topics of conversation around cultured meat and raised new research questions. Based on analysis conducted using social network analysis methods, the value propositions that are most commonly communicated by users were identified: (1) clean meat, (2) future food, (3) sustainable food, and (4) alternative protein. Based on text analysis through LDA, five topics were identified as representing the main areas of discussion on Twitter. First, clean and sustainable products. This topic focuses on communicating the main values of cultured meat, which include sustainable and clean meat production and the growth of a food industry without animal cruelty. Second, the impact on agribusiness and plant-based protein. This community focuses on the impact of cultured meat on the agribusiness sector and its possibility of supplementing or replacing plant-based protein. Third, positive environmental aspects. This topic focuses on the positive effects of cultured meat in terms of reducing the energy needed for meat production and, consequently, reducing greenhouse gas emissions. Fourth, cultured meat as an alternative protein. This topic focuses on communicating the value of cultured meat as a source of better or alternative protein, which can be a product for vegans. Fifth, the regulation of cultured meat. This topic focuses on technological regulation by the USDA and FDA. These results are especially important in terms of understanding the value proposition of cultured meat on social media, which shapes the prejudices and perceptions of future consumers about cultured meat.

## Figures and Tables

**Table 1:** Frequency value of the top 20 hashtags published in connection with cultured meat on the social network Twitter.

No.	Hashtag	Frequency	No.	Hashtag	Frequency
1	#culturedmeat	6286	11	#cellularagriculture	548
2	#cultivatedmeat	3844	12	#futureoffood	542
3	#cleanmeat	1576	13	#beef	418
4	#cellag	879	14	#3dprinting	409
5	#foodtech	835	15	#bioprinting	383
6	#futurefood	744	16	#food	301
7	#cellbasedmeat	738	17	#labgrownmeat	293
8	#meat	685	18	#sustainability	267
9	#plantbased	589	19	#alternativeprotein	250
10	#cellbased	556	20	#vegan	221

**Table 2:** Eigenvector centrality values of the top 20 hashtags published in connection with cultured meat on the social network Twitter.

No.	Hashtag	Eigenvector Centrality	No.	Hashtag	Eigenvector Centrality
1	#culturedmeat	1.0	11	#sustainability	0.239394
2	#cultivatedmeat	0.519864	12	#labgrownmeat	0.237006
3	#cleanmeat	0.425025	13	#futurefood	0.234675
4	#meat	0.38388	14	#vegan	0.230525
5	#food	0.3091572	15	#futureoffood	0.221621
6	#foodtech	0.297311	16	#science	0.213005
7	#plantbased	0.284035	17	#innovation	0.206331
8	#cellbasedmeat	0.271083	18	#cellbased	0.205934
9	#cellularagriculture	0.253829	19	#alternativeprotein	0.205733
10	#cellag	0.239989320	20	#beef	0.18995

**Table 3:** Top 20 words in Tweets, based on regional difference (USA and EU).

USA	Count	EU	Count
meat	4727	meat	2268
cultured	3075	cultured	1686
cell	1373	based	546
based	1004	cultivated	390
cultivated	1002	cell	368
food	681	plant	331
clean	496	food	304
future	418	clean	221
industry	324	eat	186
grown	323	animal	180
eat	322	novel	179
animal	318	forecasts	177
via	309	future	176
plant	301	replacements	174
world	275	cells	172
market	241	forecasting	171
production	241	sustainable	164
cells	216	via	154
sustainable	208	world	151
company	208	grown	144

**Table 4:** Topics extracted based on LDA exploration.

No. *	Topic Name	Key Terms	Size (%)
1	Clean and sustainable product	Grown, food, industry, animals, production, sustainable, clean	39
2	Comparison with plant-based protein and impact on agribusiness	Food, market, future, consumers, plant, agriculture, global	28.4
3	Positive environmental aspects	Energy, gas, greenhouse	14.4
4	Cultured meat as alternative protein	Protein, better, vegan, alternative, better	11
5	Regulation of cultured meat	USDA, FDA, technology, produce	7.2

* The numbers relate to Figure 3.

## Data Availability

All data used in this study can be downloaded via Twitter API [77].
